# Inhibition of Sonic Hedgehog Signaling Suppresses Glioma Stem-Like Cells Likely Through Inducing Autophagic Cell Death

**DOI:** 10.3389/fonc.2020.01233

**Published:** 2020-07-24

**Authors:** Hui-Chi Hung, Chan-Chuan Liu, Jian-Ying Chuang, Chun-Lin Su, Po-Wu Gean

**Affiliations:** ^1^Department of Pharmacology, College of Medicine, National Cheng-Kung University, Tainan, Taiwan; ^2^Institute of Basic Medical Sciences, College of Medicine, National Cheng-Kung University, Tainan, Taiwan; ^3^Graduate Institute of Neural Regenerative Medicine, College of Medical Science and Technology, Taipei Medical University, Taipei, Taiwan; ^4^Division of Natural Sciences, Center for General Education, Southern Taiwan University of Science and Technology, Tainan, Taiwan; ^5^Department of Biotechnology and Bioindustry Sciences, National Cheng-Kung University, Tainan, Taiwan

**Keywords:** sonic hedgehog, glioblastoma, stem-like cells, autophagy, amiodarone

## Abstract

Glioblastoma (GBM) often recurs after radio- and chemotherapies leading to poor prognosis. Glioma stem-like cells (GSCs) contribute to drug resistance and recurrence. Thus, understanding cellular mechanism underlying the growth of GSCs is critical for the treatment of GBM. Here GSCs were isolated from human U87 GBM cells with magnetic-activated cell sorting (MACS) using CD133 as a marker. The CD133^+^ cells highly expressed sonic hedgehog (Shh) and were capable of forming tumor spheroids *in vitro* and tumor *in vivo*. Athymic mice received intracranial injection of luciferase transduced parental and CD133^+^ GBM cells was utilized as orthotopic GBM model. Inhibited Shh by LDE225 delayed GBM growth *in vivo*, and downregulated Ptch1 and Gli1. CD133^+^ cell proliferation was more sensitive to inhibition by LDE225 than that of CD133^−^ cells. Treatment with LDE225 significantly reduced CD133^+^-derived tumor spheroid formation. Large membranous vacuoles appeared in the LDE225-treated cells concomitant with the conversion of LC3-I to LC3-II. In addition, LDE225-induced cell death was mitigated in the presence of autophagy inhibitor 3-methyladenine (3-MA). Tumor growth was much slower in *Shh* shRNA-knockdown mice than in control RNA-transfected mice. Conversely, tumor growth was faster in Shh overexpressed mice. Furthermore, combination of LDE225 and rapamycin treatment resulted in additive effect on LC3-I to LC3-II conversion and reduction in cell viability. However, LDE225 did not affect the phosphorylated level of mTOR. Similarly, amiodarone, an mTOR-independent autophagy enhancer, reduced CD133^+^ cell viability and tumor spheroid formation *in vitro* and exhibited anti-tumor activity *in vivo*. These results suggest that Shh inhibitor induces autophagy of CD133^+^ cells likely through mTOR independent pathway. Targeting Shh signal pathway may overcome chemoresistance and provide a therapeutic strategy for patients with malignant gliomas.

## Introduction

Glioblastoma (GBM) is one of the most common and malignant subtype of brain tumors in adults (1). Although the incident rate is relatively low, GBM is very invasive that leads to rapid neurological destruction and a disproportionately high mortality. Despite the improvement of new surgical and radiation techniques, the median survival rate of patients with GBM is rather low (2, 3). A blood-brain-barrier permeable DNA alkylating agent temozolomide (4) is currently the first line chemotherapy for treating GBM. In clinics, combination of radiotherapy with temozolomide significantly prolongs the survival rate for GBM patients (5, 6). Unfortunately, tumor often regrows after radio- and chemotherapies (7, 8) resulting in poor prognosis (9, 10). The recurrence is caused at least in part, by the resistance of GBM to conventional chemo- and radio-therapies (11–14), highlighting an urgent necessity of designing new strategies for the treatment of GBM.

Sonic hedgehog (Shh) is a member of the hedgehog (Hh) family which functions as a chemical signal in transmitting information to the embryonic cells required for normal development. Shh plays a critical role in the regulation of vertebrate organogenesis and the development of brain and spinal cord including midbrain and ventral forebrain neuronal differentiation and proliferation, and many other parts of the body (15–18). Because of its role in embryonic development, aberrant or dysregulation of Shh signaling has been implicated in the initiation and/or maintenance of different types of tumor (19–21) including GBM (22). Consistent with these reports, Shh antagonists have been shown to possess anti-tumor activity in patients with basal cell carcinoma and medulloblastoma (21, 23, 24).

Cancer stem cells (CSCs) are cancer cells that possess the ability to replenish tumors through the self-renewal and differentiation into multiple cell types (25, 26). CSCs have been identified in the breast, colon, brain and other areas and can differentiate into all the cell phenotypes of the parental tumor (27, 28). CSCs are hypothesized to be associated with chemo- and radio-resistance that lead to recurrence of tumor formation. In this theory, conventional radio- and chemotherapies kill differentiated or differentiating cells which form the bulk of the tumor, while sparing CSCs. Therefore, targeting CSCs offers a promising approach to improve cancer treatment or even cure cancer (29). In the present study, we isolated glioma stem-like cells (GSCs) from human GBM cell line U87MG (U87) using CD133 as a marker. We found that Shh expression is higher in the CD133^+^ cells than in the CD133^−^ cells. LDE225, a smoothened antagonist (30, 31), delayed GBM growth *in vivo* and significantly reduced the number of tumor spheroids derived from CD133^+^ cells. Furthermore, tumor growth was much slower in *Shh* knockdown mice suggesting that glioma growth may be dependent on a small population of CD133^+^ cells that are regulated by the Shh pathway.

## Materials and Methods

### Animals

The BALB/cAnN.Cg-Fox*n1*^*nu*^/CrlNarl mice were purchased from the National Laboratory Animal Center (NLAC). Five mice were housed in a cage with controlled temperature (22 ±2°C) and humidity (55 ± 5%), kept on a 12 h light/dark cycle, and were given free access to water and food. Care and use of laboratory animals were in accordance with National Institutes of Health (NIH) guidelines. All the procedures were approved by the Institutional Animal Care and Use Committee of the College of Medicine, NCKU, with project approval number (#104064 and #107106).

### Cell Culture

The human glioblastoma (GBM) cell lines U87MG (U87) was provided by Dr. Michael Hsiao (Genomics Research Center, Academia Sinica, Taiwan). GBM patient-derived cell line P#5 was developed by Dr. Jian Ying Chuang. Both cells were cultured in Dulbecco's Modified Eagle medium-high glucose (DMEM-high glucose, Caisson) supplemented with 10% fetal bovine serum (FBS, Sigma-Aldrich), 100 U/ml penicillin, and 0.1 mg/ml streptomycin (Caisson). All cells were maintained in a humidified incubator with 5% CO_2_ at 37°C.

### Isolation and Characterization of Cancer Stem Cells From Glioblastoma Cell Line

For magnetic-activated cell sorting (MACS) purification, fresh GBM and spheroids were dissociated, washed, and incubated either with PE conjugated CD133/2 or IgG2b (Miltenyi Biotech, 1:11) at a concentration of 10^8^ nucleated cells per ml at room temperature for 15 min. EasySep^?^ PE selection cocktail at 100 μl/ml cells was added and mixed down for more than 5 min. Magnetic cell separation was performed using manual FalconTM polystyrene round-bottom tubes and an EasySep^?^ Magnet machine. Tube was removed from magnet and cells resuspended in an appropriate amount of desired medium. The CD133^+^ cells were incubated in neural stem cells selection medium (NeuroCult^TM^ NS-A Basal Medium, NeuroCult^TM^ NS-A Proliferaction Supplement, bFGF 10 ng/mL, EGF 10 ng/mL, 20 μg/mL Heparin; STEMCELL, Canada) and gave rise to non-adherent spheres on Ultra Low Attachment Multiple Well Plates (CORNING). CD133^+^ cells were initially allowed to form tumor spheroids in suspension culture, dissociated using Accutase (BD Biosciences) at 37°C for 30 min, and then split 1:3 to 1:5. The number of tumor spheroids formed by CD133^+^ cells treated with or without LDE225 (25 μM) for 7 days in culture were determined with an Olympus DP72 image analysis system and Inverted fluorescence microscope Olympus IX71.

### Cell Proliferation Assay

Cell proliferation was measured using WST1 [2-(4-iodophenyl)-3-(4-nitrophenyl)-5-(2,4-disulfophenyl)-2H-tetrazolium] assay (Clontech Laboratories, California, USA). The CD133^+^ cells were seeded at a concentration of 8,000 cells/well in 200 μl culture medium containing various concentrations of LDE225 or cyclopamine (e.g., final concentration of 1–100 μM) into 96-well plates. The cells were incubated for 48 h at 37°C and 5% CO_2_. WST-1 reagent (10 μl/well) was added, and cells were incubated for 1 h at 37°C and 5% CO_2_. The absorbance of the product was measured at 440 nm with a microplate (ELISA) reader. The cell counts were determined by the percentage of the absorption relative to the vehicle-treated control culture.

### Western Blotting Assay

Cell pellets were collected, centrifuged at 4,000 rpm and stored at −80°C. Drugs- or vehicle-treated cell pellets were lysed in a RIPA lysis buffer containing 50 mM Tris–HCl, pH 7.4, 150 mM NaCl, 1% Nonidet P-40, 0.25% sodium deoxycholate, 0.1% sodium dodecyl sulfate (SDS), protease inhibitor (Roche) and phosphatase inhibitor (Roche). Lysates were shaken at 40 rpm on ice for 1 h and then centrifuged at 12,000 rpm for 30 min at 4°C. The mouse brain tissues were homogenized in a lysis buffer (50 mM Tris-HCl, pH 7.5, 0.3 M sucrose, 5 mM EDTA and protease/phosphatase inhibitor cocktail (Roche, Nutley, USA). Supernatants were collected and then protein concentration was measured by Bradford assay. The protein was resuspended in 5X sample buffer (12.5 mM Tris, 25% glycerol, 4% SDS, 1.54% DTT, and 0.02% Bromophenol blue). The total protein was denatured at 95°C for 5 min. Protein electrophoresis on 15 or 8% SDS-polyacrylamide gel under 120 volt, and the separated protein was transferred to a PVDF membrane (Immunobilon transfer membranes, Millipore) by semi-dry transfer system (BIO-RAD) under 350 mA, 20 volt for 1.5 h. Membranes were incubated with blocking buffer (3% bovine serum albumin in PBS) for 1 h. Membranes were incubated with the following primary antibodies: rabbit anti-Shh (1:3,000, Millipore, Darmstadt, Germany), rabbit anti-P62 (1:2,000, Cell Signaling Technology, Danvers, USA), mouse anti-LC3 (1:2,000, MBL, Japan), mouse anti-CD133 (1:5,000, Millipore, Darmstadt, Germany), rabbit anti-Ptch1 (Protintech, Illinois, USA), rat anti-Gli1 (Sigma, Darmstadt, Germany) antibody overnight at 4°C, followed by treatment with HRP-conjugated secondary antibodies (Jackson ImmunoResearch Lab., West Grove, USA) for 1 h at room temperature. After three rinses with 0.3% Triton X-100 in PBS for 10 min each, the ECL-plus chemical reagents (PerkinElmer) were added to the membrane. Films (Fuji, Japan) were exposed at different time points to ensure the optimum density. The band intensities were analyzed for densitometry by using Windows Image-J version 1.29. The protein levels in all groups were expressed as a percentage of those in controls.

### Immunofluorescent Staining

For immunostaining of tumor spheroids, CD133^+^ cells were initially allowed to form spheroids in suspension culture for 7 days. The cells were then fixed with 4% paraformaldehyde in PBS pH 7.4 for 15 min at room temperature. The cells were then incubated for 1 h in blocking solution (1% bovine serum albumin), and then incubated in primary antibodies at 4°C overnight. The following primary antibodies were used: mouse anti-CD133 (1:100, Millipore, Darmstadt, Germany), rabbit anti-SOX-2 (1:1,000, Abcam, Cambridge, UK) and rabbit anti-Shh (1:200, Millipore, Darmstadt, Germany), rabbit anti-ALDH1A1 (Protintech, Illinois, USA), rat anti-ABCG2 (Santa Cruz, Texas, US), rabbit anti-LC3 (Novus, Colorado, US). After three rinses with PBS containing 0.1% Triton X-100 for 10 min each, the cells were incubated in secondary antibodies at room temperature for 1 h. The following secondary antibodies were used: Alexa Fluor®594-conjugated Goat-anti-rabbit IgG (Jackson ImmunoResearch Lab., West Grove, USA) and Alexa Fluor®488-conjugated Sheep-anti-mouse IgG and Alexa Fluor®488-conjugated Goat-anti-rat IgG (Jackson ImmunoResearch Lab., West Grove, USA). DAPI (4',6-diamidino-2-phenylindole) (1:1,000, Sigma-Aldrich, St. Louis, USA) dye was used as nuclear counterstain. The cells are washed in PBS containing 0.1% Triton X-100 three times for 10 min. The sections were cover-slipped with Prolong^?^ gold anti-fade reagent (Life Technologies, Carlsbad, USA). Fluorescence signals were detected with a Leica DM 2500 microscope and MetaMorph image software used for counting the cells number.

### JC-1 Mitochondrial Membrane Potential Assay

Early apoptosis was measured by using JC-1 Mitochondrial Membrane Potential assay (Cayman Chemical, Michigan, USA). The CD133^+^ cells were seeded at a concentration of 5,000 cells/well in 100 μl culture medium containing 25 μM LDE225 or 0.1% DMSO as vehicle control into black 96-well plates. The cells were incubated for 48 h at 37°C and 5 % CO_2_. JC-1 Staining solution (10 μl/well) was added, and cells were incubated for 30 min at 37°C and 5% CO_2_. Plates were centrifuged at 300 × g at room temperature. The supernatant was removed, and JC-1 buffer was used to suspend JC-1 stained cells. The fluorescence intensity of J-aggregate and JC-1 monomer was measured with excitation and emission at 535/595 nm and 485/535 nm with a microplate (ELISA) reader. The ratio of J-aggregate and JC-1 was determined as early apoptosis.

### Caspase-Glo® 3/7 Assay

The activity of caspase 3 and 7 was measured by using Caspase-Glo® 3/7 Assay (Promega, Wisconsin, US). The CD133^+^ cells were seeded at a concentration of 1,000 cells/well in 100 μl culture medium containing 25 μM LDE225 or 0.1% DMSO as vehicle control into white 96-well plates. The cells were incubated for 48 h at 37°C and 5% CO_2_. Caspase-Glo®3/7 Reagent (10 μl/well) was added, and cells were incubated for 1 h at 37°C and 5% CO_2_. The luminescence was recorded with a microplate (ELISA) reader. The activity of caspase 3 and 7 was determined by the percentage of the luminescence signal relative to the control culture.

### *In vivo* Intracranial Xenograft Animal Model and Bioluminescence Imaging

U87 GBM cells were transduced with lentiviral vector expressing GFP and firefly luciferase. GFP/Luc expressing cells were sorted out for further passages (FACS-Aria, BD Biosciences). For tumorigenesis, luciferase-expressing GBM cells were inoculated intracranially into the 8- to 10-week-old male nude mice (BALB/cAnN-Foxnlnu/CrlNarl mice, National Laboratory Animal Center). Nude mice were anesthetized with chloral hydrate and placed on a stereotaxic device. Subsequently, a hamilton syringe with 30-gauge needle was mounted on a stereotaxic device, and luciferase-expressing GBM cells were injected into the left side of the brains, 1.5 mm caudal and lateral to the bregma, and at a depth of 3.5 to 4 mm. LDE225 (Cayman) was injected intraperitoneally injected at a dose of 20 mg/kg twice weekly. Tumor growth was monitored by IVIS spectrum Live Imaging System (IVIS-200, Xenogen) twice weekly. Before monitoring, mice were injected with 150 mg/kg D-luciferin (PerkinElmer), and simultaneously anesthetized with isoflurane. The results of luciferase radiance were quantitated by Live Imaging Software (Xenogen) and the results were analyzed by using GraphPad Prism software.

### *Shh* shRNA Lentivirus Production

Production of lentivirus was initiated by triple transfection of HEK293T cells by a Lipofectamine® LTX Reagent (Life Technologies, Carlsbad, USA) method using small hairpin interfering RNA (shRNA) together with pCMV-dR8.91 and pMD2.G. The *Shh*-shRNA or scrambled shRNA conjugated on the vector of pLKO.1 with puromycin-resistant region was provided by National RNAi Core Facility (Institute of Molecular Biology, Academia Sinica, Taiwan). Cells were harvested 48 h later, medium containing lentiviruses filtered with 0.45 μm filters and viral particles were concentrated from the supernatant by Lenti-X™ Concentrator (Clontech Laboratories, Mountain View, USA) and purified to yield 1 × 10^8^ transducing units/ml storing at −80°C until use. The target sequence of *Shh*-shRNA is described as follows: *Shh*-shRNA: 5′-GCGGAAGGTATGAAGGGAAGA-3′.

### *Shh*-Over-Expression

To create lentiviruses over-expressing *Shh*, full-length *Shh* open reading frames (ORFs) (NM_000193; GenScript, New Jersey, USA) was amplified by PCR and was inserted into pLVX-IRES-ZsGreen1 expression vector (Clontech Laboratories, California, USA). The pLVX-NES1-IRES-ZsGreens1 vector encoding *Shh* (or empty vector) and the two packaging plasmids (pCMV-dR8.91 and pMD2.G) were co-transfected into HEK293T cells by lipofectamine® LTX Reagent (Life Technologies, Carlsbad, USA). Lentiviruses were harvested at 48 h after transfection, filter lentivirus supernatant through a 0.45 μm PVDF membrane filters, concentrated by Lenti-X™ Concentrator (Clontech Laboratories, Mountain View, USA), purified to yield 1 × 10^8^ transducing units/ml and stored at −80°Cuntil use.

### Statistical Analysis

Experiments were performed at least in triplicate. All results were presented as mean ± standard error of the mean (SEM). Independent experiments were analyzed by unpaired *t*-test. Two-way ANOVA was used to analyze the differences in tumor spheroids numbers *in vitro* and intracranial tumor growth *in vivo* at different times of treatment. Levels of *p* < 0.05 were considered to be of statistical significance.

## Results

CD133 has been reported as a marker of human neural and brain tumor stem cells (32, 33). We previously have isolated cancer stem-like cells with CD133 from glioblastoma (GBM) cell lines using magnetic bead cell sorting (34). Then CD133^+^ and CD133^−^ cell populations were collected and cultured separately.

### Shh Expression Is Higher in CD133^+^ Cells

**Figure 1:**
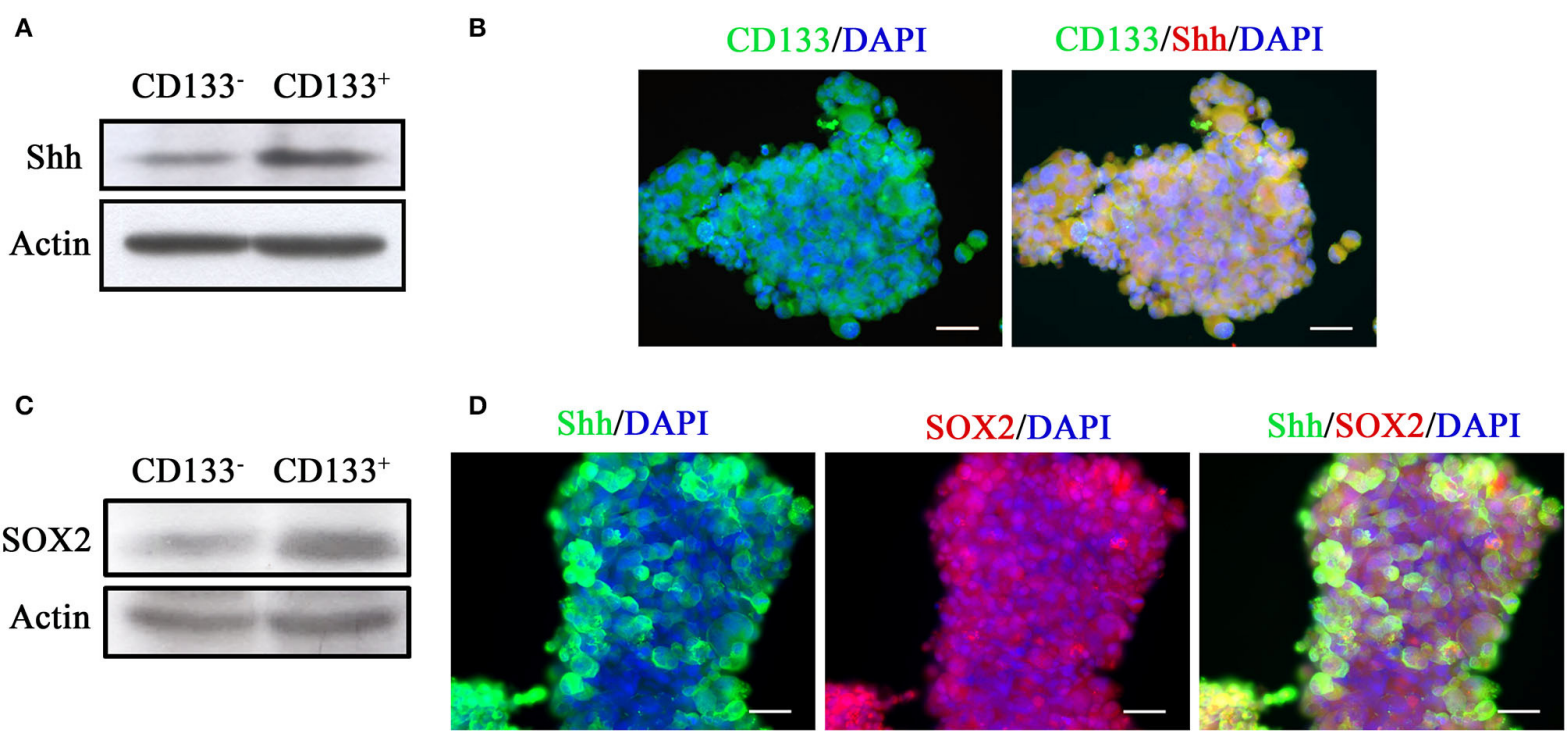
Tumor spheroids derived from CD133^+^ cells exhibit higher level of Shh expression. **(A)** Expression of Shh in CD133^+^ and CD133^−^ cells determined by Western blotting analysis. The CD133^+^ cells exhibited higher level of Shh expression. **(B)** Immunofluorescence staining of CD133 and Shh in tumor spheroids derived from CD133^+^ cells. CD133 and Shh were co-localized at tumor spheroids. **(C)** Expression of SOX2 in CD133^+^ and CD133^−^ cells determined by Western blotting analysis. The CD133^+^ cells exhibited higher level of SOX2 expression. **(D)** Immunofluorescence staining of CD133, Shh, and SOX2 in tumor spheroids derived from CD133^+^ cells. CD133 and SOX2, Shh, and SOX2 were co-localized at tumor spheroids.

We examined Shh expression in CD133^+^ and CD133^−^ cells. Western blotting analysis showed that Shh expression was higher in the CD133^+^ cells than in the CD133^−^ cells (Figure 1A). Immunofluorescence staining revealed that most of CD133^+^ cells were positive and co-localized with Shh (Figure 1B). SRY (sex determining region Y)-box 2 (SOX2) is a transcription factor that plays an important role in maintaining embryonic and neural stem cells (35, 36). Figure 1C showed that SOX2 expression was higher in the CD133^+^ cells than in the CD133^−^ cells.These Shh-positive cells were also co-immunolabeled with SOX2 (Figure 1D). ABCG2 is a member of the ATP-binding cassette (ABC) transporter superfamily. The expression level of ABCG2 has been implicated in multidrug resistance (MDR) in cancer chemotherapy and the ability of self-renewal which correlates with CD133 (37, 38). AlDH1A1 is a member of the highly conserved ALDH family that is observed in several cancer stem cells, and is often used to isolate and functionally characterize cancer stem cells (39). We also examined the expression of ABCG2 and AlDH1A1 by immunofluorescence in parental and CD133^+^ cells. The expression of ABCG2 and ALDH1A1 is higher in CD133^+^ cells than in parental cells (Supplemental Figure 1).

**Figure 2:**
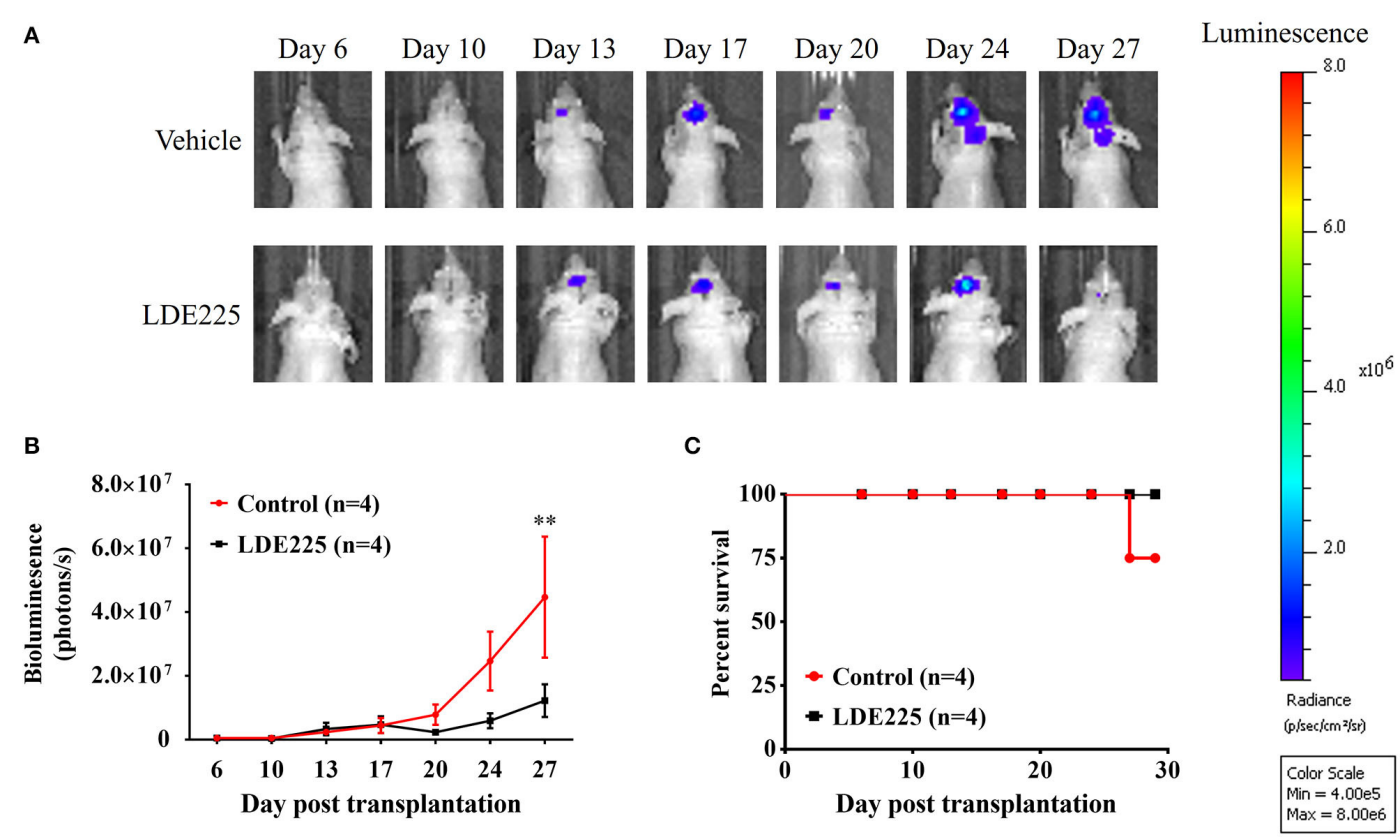
LDE225 delays tumor growth in an intracranial tumor model. **(A)** Luc-expressing parental U87 cells (3 × 10^5^ cells) were injected intracranially into athymic mice and tumor growth was monitored using the IVIS-200 imaging system. **(B)** Tumor growth was slower in LDE225 treated mice than in vehicle control mice. **p < 0.001 vs. vehicle control. **(C)** Kaplan–Meier analysis of LDE225 treatment on survival.

**Figure 3:**
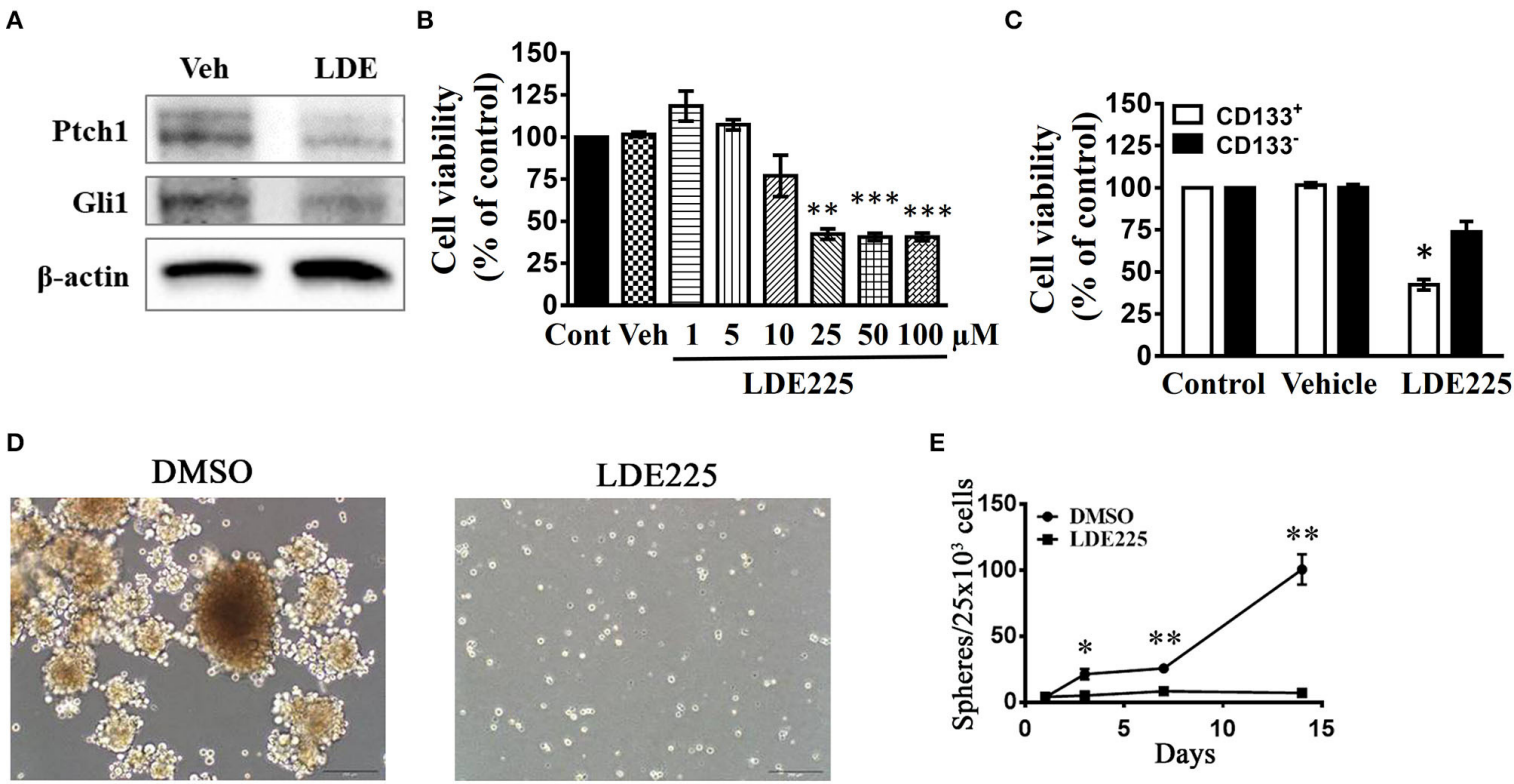
Effects of Shh inhibitors LDE225 on the cell viability of CD133^+^ and CD133^−^ cells. **(A)** CD133^+^ cells were treated with LDE-225 (25 μM) for 48 h and the expression of Patched1 and Gli1 was determined by the Western blotting analysis. LDE225 downregulated Patched1 and Gli1 in CD133^+^ cells. **(B)** Concentration-dependent effect of LDE225 on the cell viability of CD133^+^ cells. **p < 0.01, ***p < 0.001 vs. vehicle. **(C)** CD133^+^ and CD133^−^ cells were treated with LDE225 (25 μM) for 48 h and cell viability was assessed using WST-1 assay. CD133^+^ cells were more sensitive to LDE225 inhibition than that of CD133^−^ cells. *p < 0.05 vs. CD133^−^ cells. **(D,E)** Effects of LDE225 (25 μM) on the number of tumor spheroids derived from CD133^+^ cells. Primary tumor spheroids derived from CD133^+^ were dissociated and cultured. They were then treated with LDE225 (25 μM) for the times as indicated in **(E)**. The medium was replaced every 2 days in the presence of LDE225. *p < 0.05, **p < 0.01 vs. Vehicle.

We used a potent and selective smoothened antagonist LDE225 (30) to determine the requirement of Shh signaling in the proliferation and tumor growth of GBM cell lines. We first confirmed the effect of LDE225 on tumor growth by using intracranial injection model. Luc-expressing parental U87 cells were injected intracranially into athymic mice and tumor growth was monitored using IVIS-200 imaging system. At day 7, LDE225 or DMSO as vehicle was injected intraperitoneally twice per week (20 mg/kg) into the mice and tumor growth was observed for 22 more days. Comparing with vehicle control, Figure 2A showed that LDE225 was able to delay tumor growth. A two-way ANOVA revealed a main effect of group (LDE225 vs. control) [*F*_(1, 42)_ =6.126, *p* < 0.05], interaction [*F*_(6, 42)_ = 2.297, *p* = 0.0524] and days after application [*F*_(6, 42)_ = 5.849, *p* < 0.001] (Figure 2B). Kaplan–Meier analysis of the survival data of vehicle control and LDE225-treated mice displayed in Figure 2C. We also confirmed whether LDE225 inhibits Shh signal pathway. CD133^+^ cells were treated with LDE225 (25 μM) or vehicle for 48 h and the expression of Patch1 and Gli1 were determined by Western blotting analysis. As shown in Figure 3A, LDE225 downregulated the expression of PATCH1 and GLI1. Next, CD133^+^ and CD133^−^ cells were treated with LDE225 (25 μM) for 48 h and cell viability was assessed using WST-1 assay. Concentration dependent relationship estimated the IC_50_ of about 20 μM for LDE225 to reduce CD133^+^ cell viability (Figure 3B), while IC_50_ of LDE225 was about 50 μM for parental cells (Supplemental Figure 2A). As shown in Figure 3C, CD133^+^ cells were more sensitive to LDE225 inhibition than that of CD133^−^ cells. At the concentration of 25 μM, LDE225 inhibited the proliferation of CD133^+^ and CD133^−^ cells by 57.57 ± 3.17% (*n* = 3) and 26.18 ± 6.22% (*n* = 4), respectively [*t*_(5)_ = 4.013, *p* < 0.05]. We also determined the effect of LDE225 on patient-derived cell line P#5 which displays various characteristics of GBM (40, 41). As illustrated in Supplemental Figure 2B, LDE225 inhibited cell viability with IC_50_ ~423 μM. Similar inhibition of CD133^+^ was observed by another Shh inhibitor cyclopamine, which binds to and inactivates smoothened protein (42) (IC_50_ ≈ 100 μM, Supplemental Figure 2C). We determined the effect of Shh inhibition on self-renew capacity of CD133^+^ cells (Figure 3D). CD133^+^ cells were treated with LDE225 (25 μM) or vehicle and CD133^+^-derived tumor spheroids were counted at 1, 3, 7, and 14 days. Treatment with LDE225 significantly reduced the number of tumor spheroids. A two-way ANOVA revealed a main effect of drug (LDE225 vs. vehicle) [*F*_(1, 16)_ = 100.9, *p* < 0.001], interaction [*F*_(3, 16)_ = 44.27, *p* < 0.001] and days after application [*F*_(3, 16)_ = 44.25, *p* < 0.001] (Figure 3E).

### Inhibition of Shh Induces Autophagy in CD133^+^ Cells

**Figure 4:**
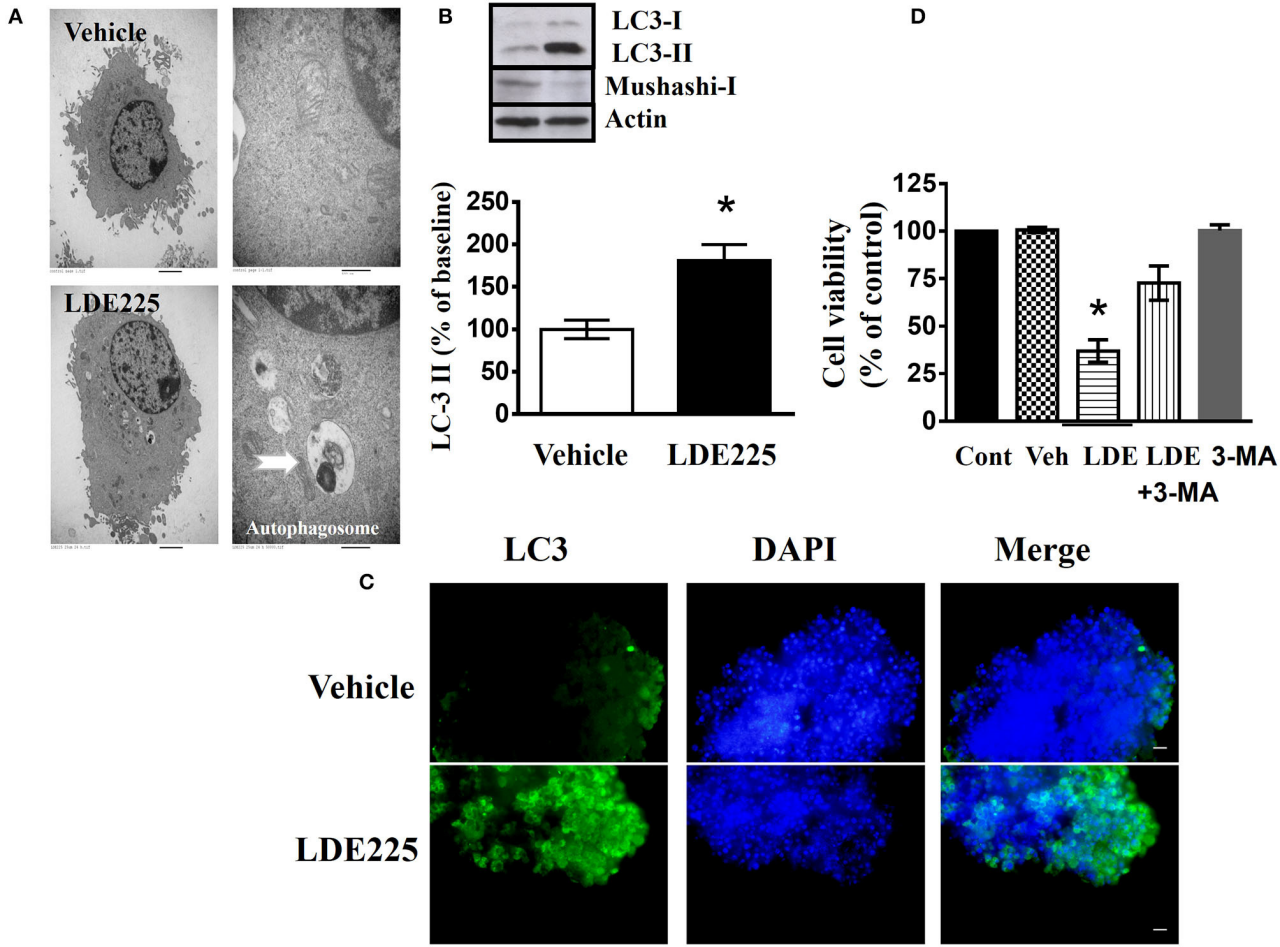
LDE225 induces autophagy in the CD133^+^ cells. **(A)** The graphs of transmission electron microscopy represented CD133^+^ cells treated with LDE225 (25 μM) or vehicle for 48 h. Arrows indicate autophagosomes. **(B)** Western blot analysis of LC3-I/LC3-II and Mushashi-1 expression in the CD133^+^ cells treated with LDE225 (25 μM) for 48 h. LDE225 induced conversion of LC3-I to LC3-II which was accompanied by the downregulation of Mushashi-1. **(C)** LC3 expression in CD133^+^ cells treated with LDE225 (25 μM) or vehicle for 48 h. The fluorescence intensity of LC3 punctate increased after LDE225 treatment. **(D)** Block of LDE225-induced cell death by autophagy inhibitor 3-MA. CD133^+^ cells were pretreated with 3-MA (3 μM) 1 h before the exposure to LDE225 (25 μM). Pretreatment with 3-MA attenuated LDE225-induced cell death. *p < 0.05.

In LDE225-treated cells, the observation by transmission electronic microscope showed the appearance of large membranous vacuoles in the cytoplasm which is a characteristic feature of cells undergoing autophagy (Figure 4A). In addition, LC3 is distributed in the autophagosome membrane (43). The conversion of LC3-I to LC3-II is a common biomarker for autophagy activation (44, 45). We determined the conversion of LC3-I to LC3-II with anti-LC3 antibody. Immunoblotting using lysates from LDE225 (25 μM)-treated CD133^+^ cells revealed a significant increase in processed LC3-II [*t*_(5)_ = 3.39, *p* < 0.05] (Figure 4B). This was accompanied by the reduced expression of Mushashi-1, a RNA–binding protein selectively expressed in neural progenitor cells (46). The intensity of LC3 fluorescence punctate also increased after LDE225 treatment (Figure 4C). Furthermore, LDE225-induced cell death was rescued by autophagy inhibitor 3-methyladenine (3-MA) (47) (Figure 4D) suggesting that autophagy plays a critical role in LDE225-induced cytotoxicity in CD133^+^ cells.

We performed different apoptosis assays to evaluate whether LDE-induced cell death involves apoptosis. We used JC-1 mitochondrial membrane potential assay which measures the mitochondrial membrane potential as the indicator of cell health. Change in fluorescent property of JC-1 dye can be utilized to evaluate early apoptosis. CD133^+^ cells were treated with LDE225 (25 μM) or 0.1% DMSO as vehicle control for 48 h. Supplemental Figure 3A shows that vehicle led to mitochondrial depolarization and decreased the ratio of J-aggregate/JC-1 monomer while that was not different between vehicle and LDE225 (25 μM) treatment. Caspase-Glo assay also revealed that Caspase 3/7 activity was not different between LDE225 and vehicle (Supplemental Figure 3B). These results suggest that LDE225-induced cell death likely was not mediated primarily through apoptosis.

### Knockdown of Shh Slows Tumor Growth in an Intracranial Tumor Model

**Figure 5:**
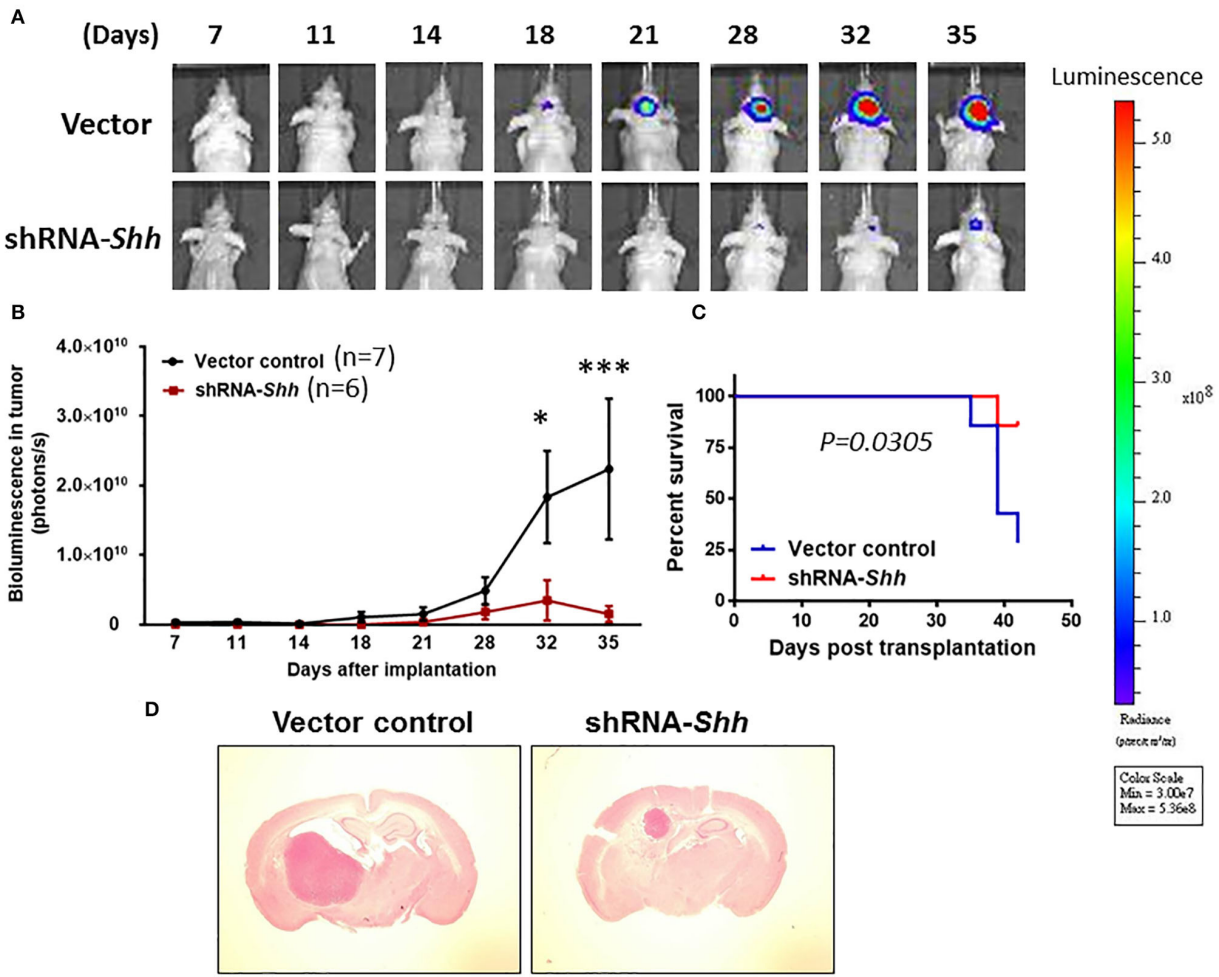
Knockdown of Shh attenuates tumor growth in an intracranial tumor model. **(A)** Luc-expressing CD133^+^ cells were infected with lentiviruses carrying the expression vector containing *Shh* shRNA. Transduced CD133^+^ cells (8 × 10^4^ cells) were injected intracranially into athymic mice and tumor growth was monitored using the IVIS-200 imaging system. **(B)** Tumor growth was much slower in *Shh* shRNA- transfected mice than in control RNA-transfected mice. *p < 0.05, ***p < 0.001 vs. control RNA-transfected. **(C)** Kaplan–Meier analysis of *Shh* shRNA transfection on survival. **(D)** Brains of mice harboring tumors generated from control RNA-transfected mice and *Shh* shRNA-transfected mice and stained by H&E. Note that tumor in *Shh* shRNA-transfected mice was significantly larger than control RNA-transfected tumor.

We clarified whether Shh plays a role in tumor growth under *in vivo* conditions using an orthotopic GBM model. To monitor intracranial tumor growth, we infected Luc-expressing CD133^+^ cells with lentiviruses carrying the expression vector containing *Shh* shRNA. Transduced CD133^+^ cells (8 × 10^4^ cells) were injected intracranially into athymic mice and tumor growth was monitored using IVIS-200 imaging system. Figure 5A shows that tumor growth was much slower in *Shh* shRNA-knockdown mice than in control RNA-transfected mice. A two-way ANOVA revealed a main effect of group (*Shh* shRNA vs. control) [*F*_(1, 11)_ = 4.980, *p* < 0.05], interaction [*F*_(7, 77)_ = 3.110, *p* < 0.01] and days after application [*F*_(7, 77)_ = 4.853, *p* < 0.001] (Figure 5B). Kaplan–Meier analysis of the survival data demonstrated a statistically significant difference (*p* < 0.05) in the median survival between vector control and *Shh* shRNA-treated mice (Figure 5C). Brain tumor growth was analyzed in coronal brain slices by H&E staining. As shown in Figure 5D, in comparison with vector control, shRNA-*Shh*-transfection generated significantly smaller intracranial tumor.

To examine whether knockdown of Shh affected autophagy and stemness *in vivo*, we examined the expression of LC3-II in *Shh* shRNA-treated mice. Supplemental Figure 4 shows that the levels of Shh, CD133, mushashi-1 and SOX2 were lower whereas the conversion of LC3-I to LC3-II was higher in mice of Shh knockdown CD133^+^ cells compared to CD133^+^ cells of control mice. These results suggest that knockdown of *Shh* reduces stemness and GBM tumor growth.

### Over-Expression of Shh Promotes Tumor Growth

**Figure 6:**
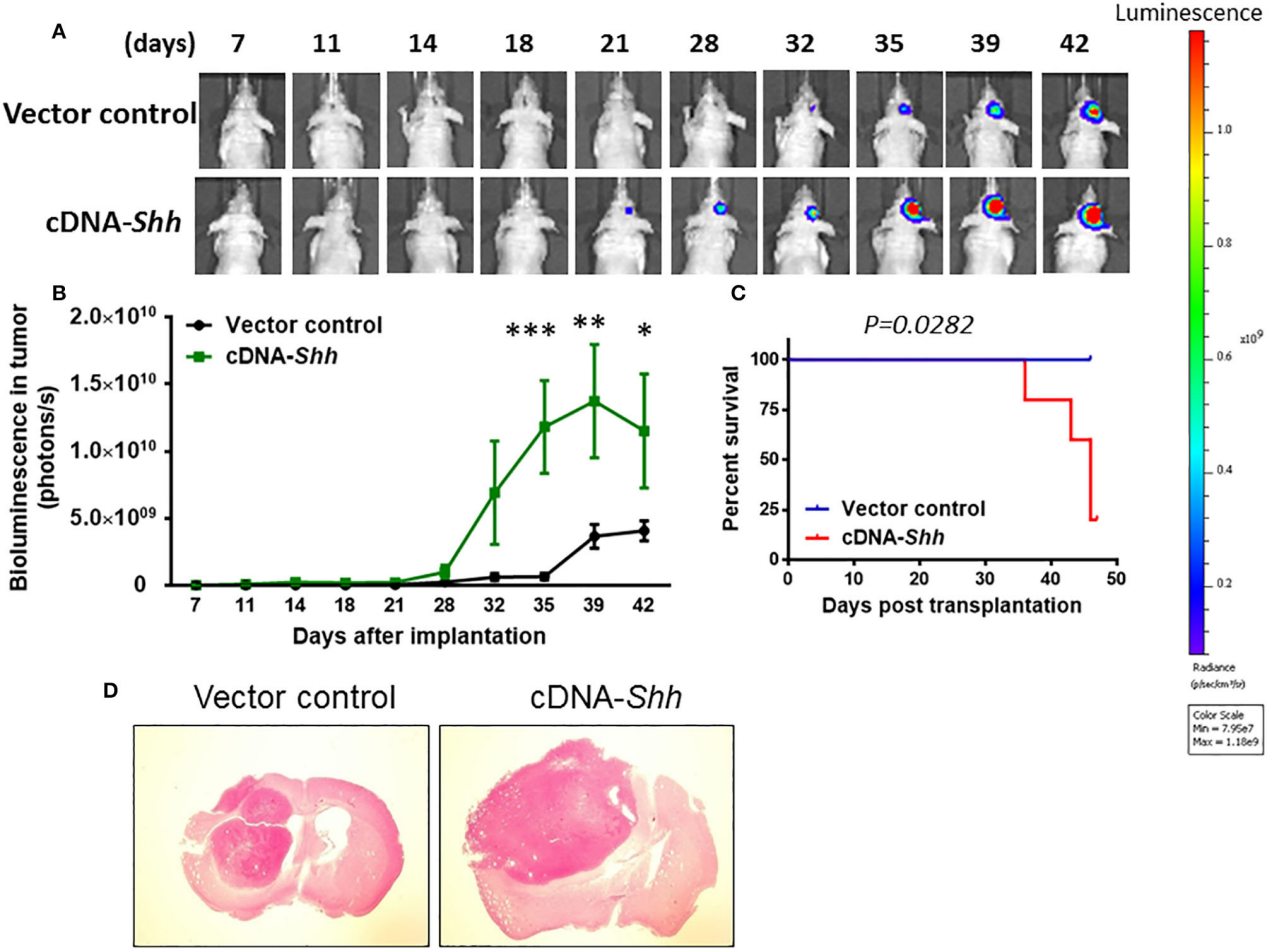
Over-expression of Shh promotes tumor growth. **(A)** Shh overexpression in parental U87 cells was transduced by lentiviruses carrying the expression vector containing mouse *Shh* gene (LV-Shh). The empty vector (LV-Vehicle) served as control. The LV-Shh or LV-Vehicle infected U87 (8 × 10^4^ cells) was injected intracranially into athymic mice. Tumor growth was monitored by IVIS-200 imaging system. **(B)** Tumor growth was much faster in LV-*Shh*-transfected mice than in LV-vehicle-transfected mice. *p < 0.05, **p < 0.01, ***p < 0.001 vs. vector control. **(C)** Kaplan–Meier analysis of over-expression of Shh transfection on survival. **(D)** Brains of mice harboring tumors generated from LV- Vehicle and LV-*Shh*-transfection and stained by H&E. Note that LV-*Shh*-transfection tumor was significantly larger than vector control tumor.

To over-express Shh, we cloned the mouse *Shh* gene into parental U87 cells with lentiviruses carrying the expression vector containing *Shh* gene. The empty vector (LV-Vehicle) served as control. The LV-Shh or LV-vehicle transfected parental U87 GBM cells (8 × 10^4^ cells) was injected intracranially into athymic mice. As shown in Figures 6A,B, tumor growth was much faster in LV-*Shh*-transfected mice than in LV-vehicle-transfected mice. A two-way ANOVA revealed a main effect of group (LV-*Shh* vs. control) [*F*_(1, 6)_ = 7.665, *p* < 0.05], interaction [*F*_(9, 54)_ = 4.022, *p* < 0.001] and days after application [*F*_(9, 54)_ = 9.691, *p* < 0.001]. Kaplan–Meier analysis of the survival data demonstrated a statistically significant difference (*p* < 0.05) in median survival between control and LV-*Shh*-treated mice (Figure 6C). Brain tumor size was analyzed in coronal brain slices. As shown in Figure 6D, in comparison with vector control, LV-*Shh*-transfection generated significantly larger intracranial tumor.

To examine whether over-expression of Shh affected autophagy and stemness *in vivo*, we examined the expression of LC3-II in LV-*Shh*-treated mice. Supplemental Figure 5 shows that the levels of Shh, CD133, mushashi-1 and SOX2 were higher whereas the conversion of LC3-I to LC3-II was lower in Shh over-expression GBM compared to that of control. These results suggest that over-expression of *Shh* promotes cancer stemness and GBM tumor growth.

### Additive Effect of Rapamycin and LDE225 on The Viability of CD133^+^ Cells

**Figure 7:**
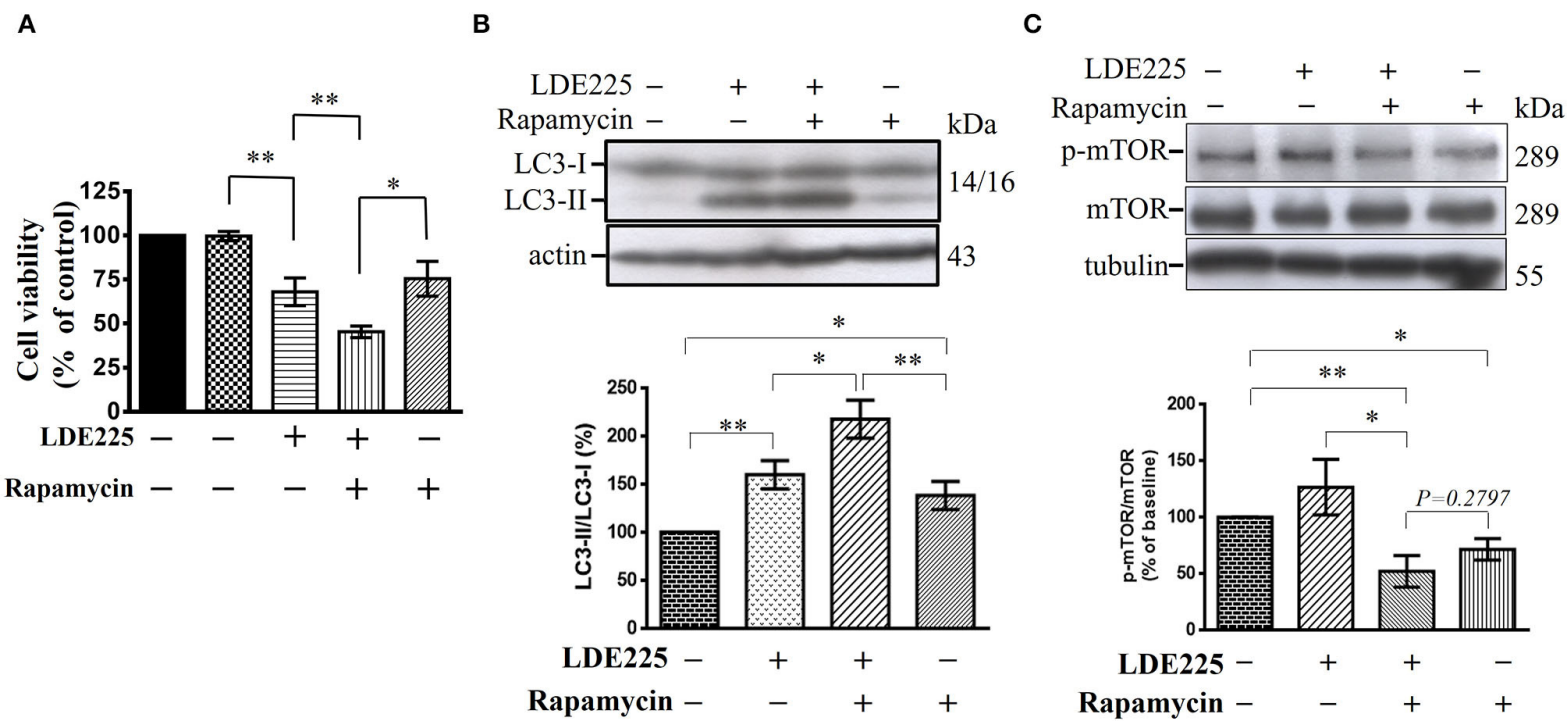
LDE225 and rapamycin in combination additively reduces cell viability and induces conversion of LC3-I to LC3-II in CD133^+^ cells. **(A)** CD133^+^ cells were treated with LDE225 (25 μM) and rapamycin (100 nM) alone or in combination for 48 h and cell viability was assessed using WST-1 assay. **(B)** CD133^+^ cells were treated with LDE225 (25 μM) and rapamycin (100 nM) alone or in combination for 48 h and the levels of LC3-I and LC3-II were measured by Western blotting analysis. **(C)** CD133^+^ cells were treated with LDE225 (25 μM) and rapamycin (100 nM) alone or in combination for 48 h and the levels of p-mTOR and mTOR were measured by Western blotting analysis. Combined treatment with LDE225 and rapamycin reduced p-mTOR to the level not different from that treated with rapamycin alone (*p* = 0.2797). *p < 0.05, **p < 0.01.

Autophagy is negatively regulated by the mammalian target of rapamycin (mTOR) and can be induced by the mTOR inhibitor rapamycin (48–50). To confirm our hypothesis, we tested the effect of rapamycin on CD133^+^ cells. Single treatment of CD133^+^ cells with LDE225 (25 μM) or rapamycin (100 nM) inhibited the cell viability by 32.0 ± 7.9% (*n* = 6) and 24.6 ± 9.9% (*n* = 6), respectively. As shown in Figure 7A, combination of LDE225 and rapamycin resulted in 54.6 ± 3.3% (*n* = 6) inhibition (*p* < 0.05 vs. single treatment). Interestingly, combined treatment resulted in additive conversion of LC3-I to LC3-II. The conversion of LC3-I to LC3-II after LDE225 or rapamycin treatment was 159.8 ± 14.7% (*n* = 7) and 138.8 ± 14.5% (*n* = 7) of control, respectively. Combined treatment resulted in 217.7 ± 19.7% (*n* = 7) of control (*p* < 0.05 vs. LDE225, *p* < 0.01 vs. rapamycin) (Figure 7B).

Autophagy can also be induced through mTOR-independent pathway (50). We determined the effect of LDE225 (25 μM) on mTOR phosphorylation. LDE225 (25 μM) did not significantly influence the phosphorylated level of mTOR (p-mTOR) whereas rapamycin (100 nM) reduced it. Combined treatment with LDE225 and rapamycin reduced p-mTOR to the level not significantly different from treatment with rapamycin alone (*p* = 0.2797) (Figure 7C). Thus, it is likely that LDE225 induced autophagy through mTOR-independent pathway.

### Amiodarone Reduces Stem-Like Cell Viability and Inhibits Tumor Formation

**Figure 8:**
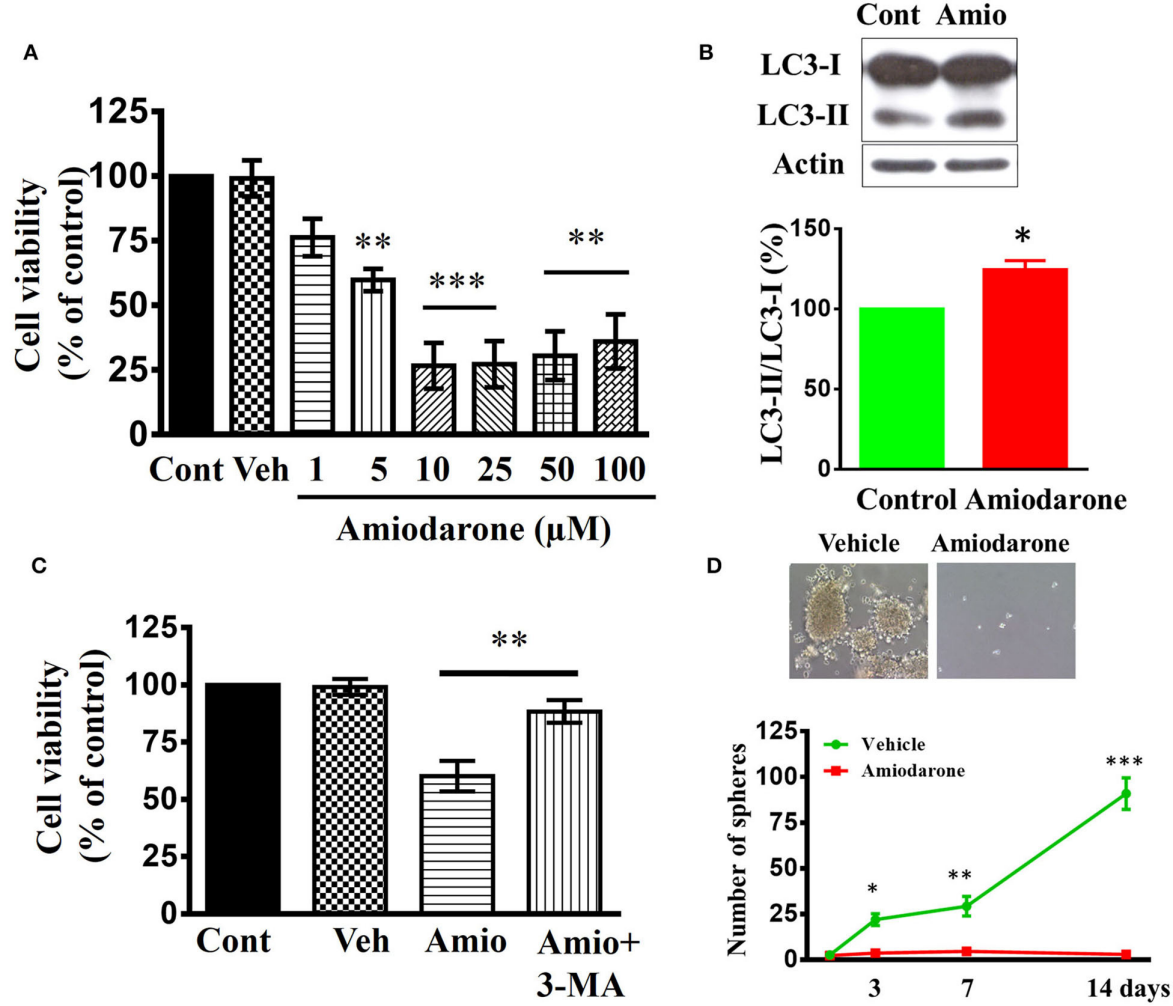
Effects of amiodarone on the cell viability and tumor spheroid formation of CD133^+^ cells. **(A)** Concentration-dependent effect of amiodarone on the cell viability of CD133^+^ cells. **p < 0.01, ***p < 0.001 vs. vehicle. **(B)** Western blot analysis of LC3-I and LC3-II expression in the CD133^+^ cells treated with amiodarone (5 μM) for 48 h. **(C)** Block of amiodarone-induced cell death by autophagy inhibitor 3-MA. CD133^+^ cells were pretreated with 3-MA (3 μM) 1 h before the exposure to amiodarone (5 μM). Pretreatment with 3-MA attenuated amiodarone-induced cell death. **p < 0.01. **(D)** Effects of amiodarone (5 μM) on the number of tumor spheroids derived from CD133^+^ cells. Primary tumor spheroids derived from CD133^+^ were dissociated and cultured. They were then treated with amiodarone (5 μM) for the times as indicated. The medium was replaced every 2 days in the presence of amiodarone. *p < 0.05, **p < 0.01, ***p < 0.001 vs. Vehicle.

Amiodarone, a clinically used anti-arrhythmic drug, could induce autophagy via mTOR-independent signaling (51). We determined whether amiodarone reduced cancer stem-like cell viability *in vitro* and exhibited anti-tumor activity *in vivo*. CD133^+^ cells were treated with amiodarone for 48 h and cell viability was assessed using WST-1 assay. As shown in Figure 8A, amiodarone dose-dependently reduced cell viability [*F*_(7, 24)_ = 16.71, *p* < 0.001]. Immunoblotting using lysates from amiodarone (5 μM)-treated CD133^+^ cells revealed a significant increase in processed LC3-II [*t*_(4)_ = 4.3976, *p* < 0.05] (Figure 8B). Furthermore, amiodarone-induced cell death was reversed by autophagy inhibitor 3-methyladenine (3-MA) (Figure 8C) suggesting that autophagy plays a critical role in amiodarone-induced cell death of CD133^+^ cells. CD133^+^ cells were treated with amiodarone (5 μM) or vehicle and CD133^+^-derived tumor spheroids were counted at 1, 3, 7, and 14 days. Treatment with amiodarone significantly reduced the number of tumor spheroids [*t*_(4)_ = 10.09, *p* < 0.001, at 14 days culture] (Figure 8D).

**Figure 9:**
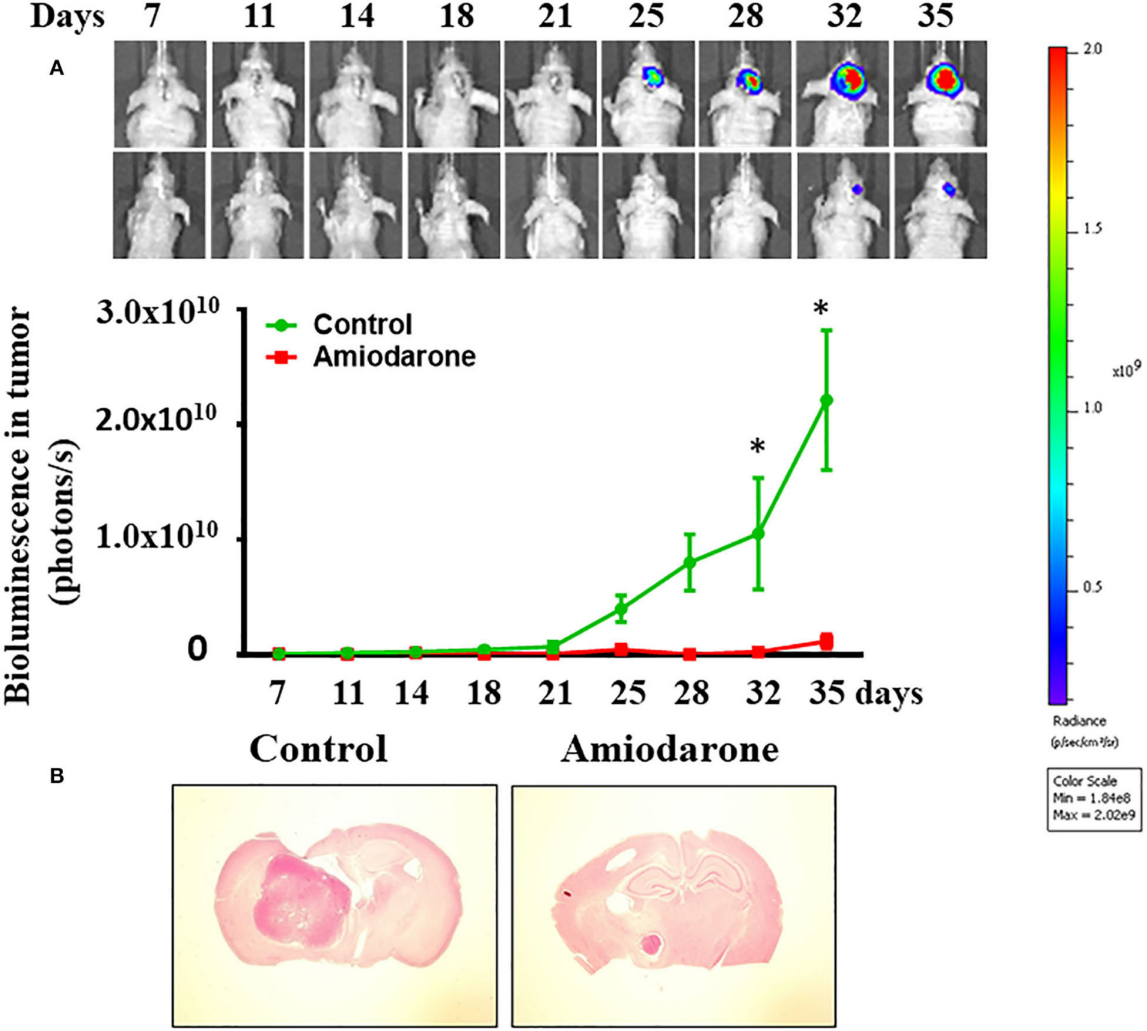
Amiodarone slows tumor growth in an intracranial tumor model. **(A)** Transduced CD133^+^ cells (6 × 10^4^ cells) were injected intracranially into athymic mice and tumor growth was monitored using the IVIS-200 imaging system. At day 11, amiodarone was injected intraperitoneally once per day for 8 days (80 mg/kg) into the mice and tumor growth was observed for 17 more days. *p < 0.05 vs. vehicle. **(B)** Amiodarone-treated mice generated significantly smaller intracranial tumor by H&E staining.

We determined whether amiodarone inhibited tumor formation. Transduced CD133^+^ cells (6 × 10^4^ cells) were injected intracranially into athymic mice and tumor growth was monitored using IVIS-200 imaging system. At day 11, amiodarone was injected intraperitoneally once per day for 8 days (80 mg/kg) into the mice and tumor growth was observed for 17 more days. Figure 9A shows that tumor growth at day 35 was much slower in amiodarone-treated mice than in control vehicle-treated mice [*t*_(8)_ = 2.758, *p* < 0.05]. Brain tumor size was analyzed in coronal brain slices. As shown in Figure 9B, in comparison with vehicle control, amiodarone-treated mice generated significantly smaller intracranial tumor.

## Discussion

### Roles of Shh Pathway in CD133^+^ Cells-Derived Tumor Spheroid Formation and Tumorigenesis

We have isolated CD133-positive cells from human U87MG (U87) GBM cells using magnetic bead cell sorting (34). CD133^+^-derived cell clones were able to grow *in vitro* in tumor spheroids and generate a tumor *in vivo* by intracranial cell injection in immunocompromised mice. The developmental signal transduction pathway mediated by Shh plays an important role during embryogenesis by regulating body patterning, cell proliferation and differentiation (52, 53). Activation of Shh has been causatively correlated with initiation and/or maintenance of cancer (54). In the present study, using Western blotting analysis and immunofluorescent staining, we showed that CD133^+^ cells exhibited higher Shh expression compared to CD133^−^ cells. Treatment with smoothened antagonist LDE225 significantly reduced the number of CD133^+^ cells-derived tumor spheroids suggesting Shh signaling likely contributes to tumor spheroids formation *in vitro*. Silencing *Shh* gene with small hairpin interfering RNA inhibited tumor growth in an intracranial mouse model. Conversely, over-expression of *Shh* gene facilitated tumor growth. Taken together, these results suggest that Shh signaling is involved in *in vitro* tumor spheroid formation and *in vivo* tumor growth.

### Shh Inhibitor Reduces the Number of CD133^+^-Derived Tumor Spheroids by Inducing Autophagy

The reports about the relationship between Shh and autophagy pathway in GBM are limited. One study in SHSY5Y cells showed that cyclopamine reduced serum starvation-induced LC3 positive cells but increased the levels of cleaved caspase 3 (54). They suggested that Shh inhibitor induced cell death by reducing autophagic processes concomitant with facilitating apoptotic cell death. In the present study, we demonstrated that LDE225 induced cell death in CD133^+^ cells in a concentration-dependent manner with IC_50_ of ~20 μM. Similar inhibition of cell survival was observed after application of another Shh inhibitor cyclopamine. Electron microscopy examination revealed that, after exposure to LDE225, CD133^+^ cells exhibited large membranous vacuoles in the cytoplasm that is a characteristic feature of cells undergoing autophagy. Further experiments of the conversion of LC3-I to LC3-II and attenuation of cell death by autophagy inhibitor 3-MA confirmed that LDE225-induced autophagy in the CD133^+^ cells. This study, in contrast to previous results, suggests that Shh inhibitor produces cytotoxicity in CD133^+^ cells by inducing autophagic cell death.

To further test this hypothesis, we used rapamycin which is a mTOR inhibitor and is capable of inducing autophagy (49, 51). We found that rapamycin reduced CD133^+^ cell viability. Interestingly, the effects of LDE225 and rapamycin were additive. Furthermore, LDE225 at the concentration that induced autophagy and reduced cell viability of CD133^+^ cells did not affect the phosphorylated level of mTOR. Combined LDE225 and rapamycin failed to show further reduction of p-mTOR compared with rapamycin alone. These results suggest that LDE225 induces autophagy through mTOR-independent pathway. These findings also provide additional support that Shh regulation of autophagy plays an important role in the survival of GSCs. Thus, Shh pathway inhibitor could act as a sensitizer to increase efficiency of conventional chemotherapeutic agents in GBM by inducing cancer stem cell autophagic death.

### Anti-tumorigenesis of Amiodarone

Amiodarone, a frequently prescribed anti-arrhythmic drugs in clinics, has been identified as a mTOR-independent autophagy enhancer (55, 56). If LDE225-induced cell death was mediated by the mTOR-independent autophagy, then amiodarone should produce similar effects as LDE225. Indeed, this was the case. Amiodarone reduced CD133^+^ cell viability and tumor spheroid formation *in vitro* and exhibited anti-tumor efficacy *in vivo*.

In summary, there was a small percentage of human U87 GBM cells which were CD133-positive, exhibited cancer stem cell markers and were capable of forming tumor spheroids *in vitro* and tumor *in vivo*. These CD133^+^ cells showed higher Shh expression and inhibition of Shh pathway with LDE225 reduced their survival and self-renew property. In LDE225-treated CD133^+^ cells, there appeared large membranous vacuoles in the cytoplasm and the conversion of LC3-I to LC3-II. LDE225-induced cell death was reversed by autophagy inhibitor 3-MA, suggesting that LDE225-induced autophagic cell death in CD133^+^ cells. Further, LDE225 did not affect the level of p-mTOR and combined LDE225 and rapamycin failed to show further reduction of p-mTOR when compared with rapamycin alone. Taken together, these results suggest that targeting Shh signal pathway may overcome chemoresistance and provide a therapeutic strategy for the treatment of malignant gliomas.

## Data Availability Statement

All datasets generated for this study are included in the article/Supplementary Material.

## Ethics Statement

The animal study was reviewed and approved by the Institutional Animal Care and Use Committee of the College of Medicine, NCKU.

## Author Contributions

J-YC, C-LS, and P-WG contributed to the conception and design of the experiments. H-CH and C-CL performed the experiments and statistical analysis. C-LS and P-WG wrote the paper. All authors contributed to the article and approved the submitted version.

## Conflict of Interest

The authors declare that the research was conducted in the absence of any commercial or financial relationships that could be construed as a potential conflict of interest.

## Supplementary Material

Supplemental Figure 1 The differential expression of CD133 and cancer stem cell markers in parental GBM cells and tumor spheroids derived from CD133^+^ cells. **(A)** Immunofluorescence staining of CD133, SOX2, ABCG2, and AlDH1A1 in parental U87 cells. **(B)** Immunofluorescence staining of ABCG2 and AlDH1A1 in tumor spheroids derived from CD133^+^ cells.

Supplemental Figure 2 The inhibitory effects of shh inhibition on cell proliferation. **(A)** Concentration-dependent effect of LDE225 on the cell viability of parental U87 cells. ^*^*p* < 0.05, ^***^*p* < 0.001 vs. vehicle. **(B)** Concentration-dependent effect of LDE225 on the cell viability of patient-derived P#5 cell line. ^*^*p* < 0.05, ^***^*p* < 0.001 vs. vehicle. **(C)** Concentration-dependent effect of cyclopamine on the cell viability of CD133^+^ cells. ^*^*p* < 0.05, ^***^*p* < 0.001 vs. vehicle.

Supplemental Figure 3 LDE225-induced cell death is not mediated primarily through apoptosis. CD133^+^ cells were treated with LDE225 (25 μM) or vehicle for 48 h. **(A)** JC-1 assay detect the changes of mitochondria potential which is able to measure early apoptosis. Vehicle (0.1% DMSO) led to mitochondrial depolarization with the decreased ratio of J-aggregate/JC-1 monomer. There was not different from LDE225 (25 μM) treatment. **(B)** Caspase/Glo assay revealed the activity of Caspase 3/7. There was not different between LDE225 and vehicle treatment.

Supplemental Figure 4 The conversion of LC3-I to LC3-II was enhanced in *Shh* shRNA transfection CD133^+^-bearing mice. Tumor tissues were collected from *Shh* shRNA or vector-control transfection CD133^+^-bearing mice. **(A)** The efficiency of *Shh* shRNA-mediated knockdown of Shh was confirmed by western blot analysis. **(B–D)** The levels of CD133 **(B)**, mushashi-1 **(C)**, and SOX2 **(D)** were lower in *Shh* shRNA transfection CD133^+^-bearing mice. **(E)** The conversion of LC3-I to LC3-II was enhanced by *Shh* shRNA transfection. ^*^*p* < 0.05 vs. control.

Supplemental Figure 5 The conversion of LC3-I to LC3-II was lower in Shh over-expression GBM-bearing mice. Tumor tissues were collected from LV-*Shh* or vector-control transfection GBM -bearing mice. **(A)** The efficiency of LV-*Shh*-mediated over-expression of Shh was confirmed by western blot analysis. **(B–D)** The levels of CD133 **(B)**, mushashi-1 **(C)**, and SOX2 **(D)** were higher in LV-*Shh* transfection GBM-bearing mice. **(E)** The conversion of LC3-I to LC3-II was reduced by LV-*Shh* transfection. ^*^*p* < 0.05, ^**^*p* < 0.01 vs. control.
