# Supplementary figures and images for: Inhibition of Sonic Hedgehog Signaling Suppresses Glioma Stem-Like Cells Likely Through Inducing Autophagic Cell Death
Source: Front Oncol. 2020 Jul 24;10:1233. doi: 10.3389/fonc.2020.01233 (PMC7393230; doi:10.3389/fonc.2020.01233)

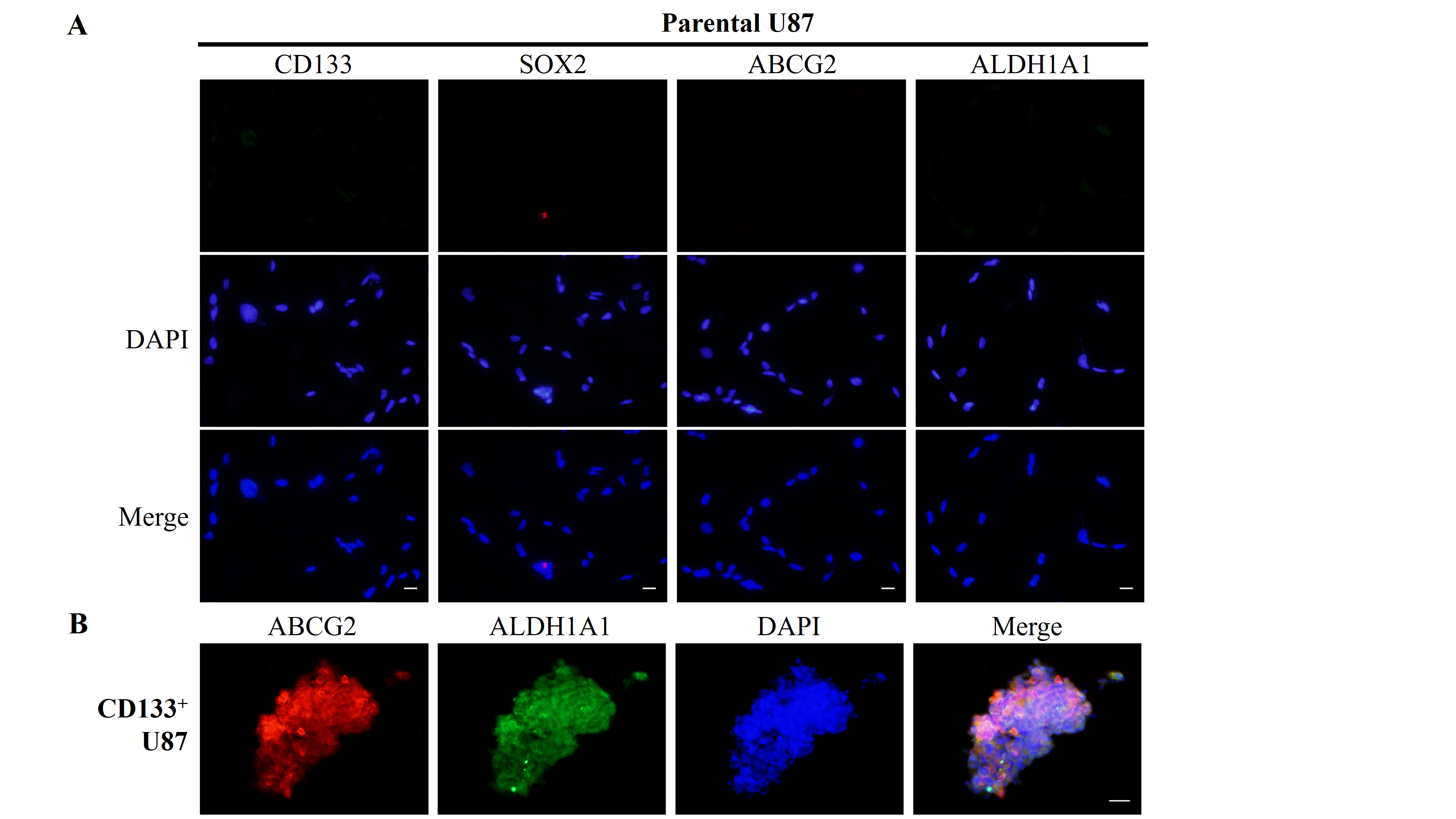

Supplement: Supplemental Figure 1 — The differential expression of CD133 and cancer stem cell markers in parental GBM cells and tumor spheroids derived from CD133+ cells. (A) Immunofluorescence staining of CD133, SOX2, ABCG2, and AlDH1A1 in parental U87 cells. (B) Immunofluorescence staining of ABCG2 and AlDH1A1 in tumor spheroids derived from CD133+ cells. [file Image_1.TIF]

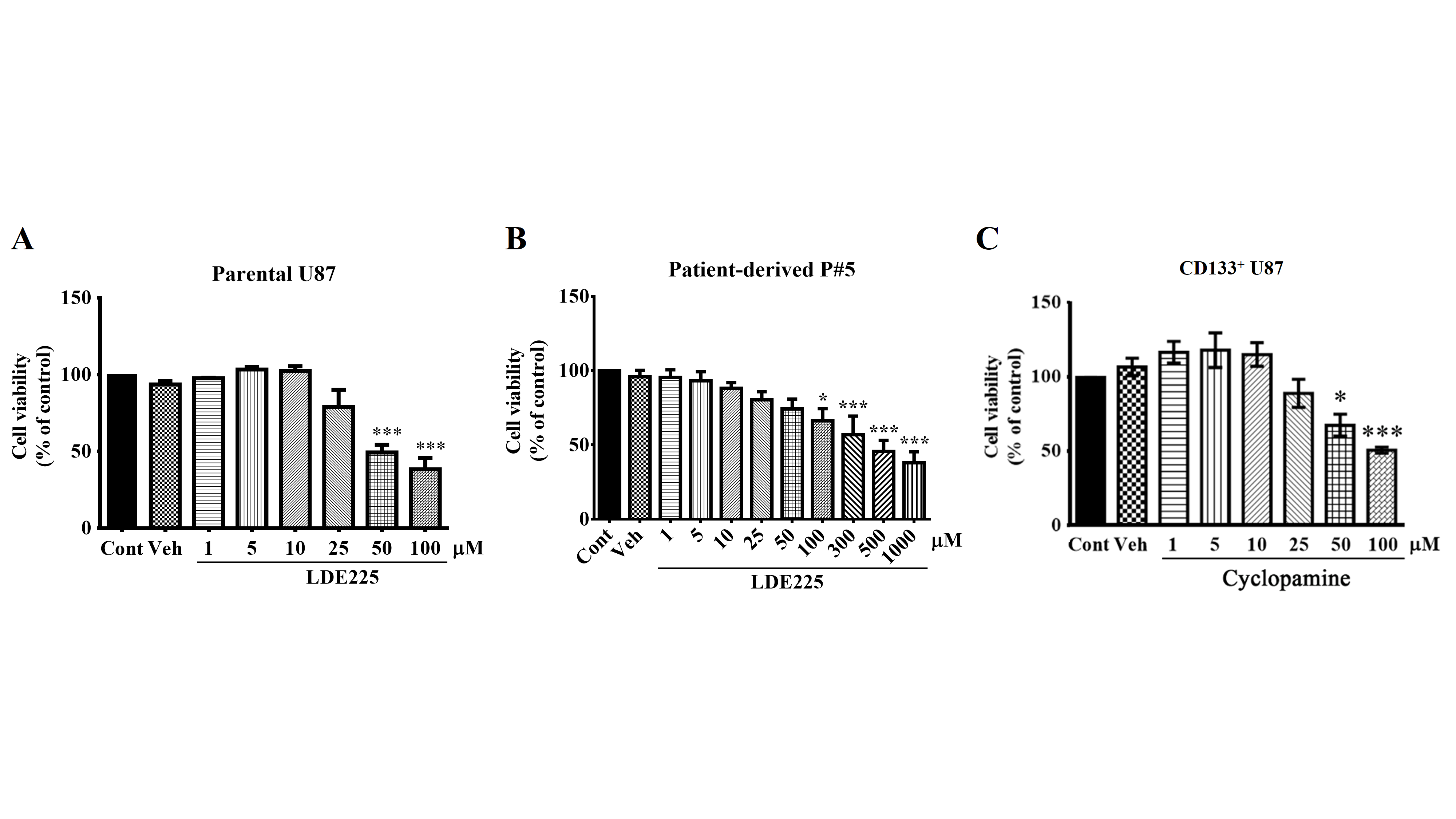

Supplement: Supplemental Figure 2 — The inhibitory effects of shh inhibition on cell proliferation. (A) Concentration-dependent effect of LDE225 on the cell viability of parental U87 cells. *p < 0.05, ***p < 0.001 vs. vehicle. (B) Concentration-dependent effect of LDE225 on the cell viability of patient-derived P#5 cell line. *p < 0.05, ***p < 0.001 vs. vehicle. (C) Concentration-dependent effect of cyclopamine on the cell viability of CD133+ cells. *p < 0.05, ***p < 0.001 vs. vehicle. [file Image_2.TIF]

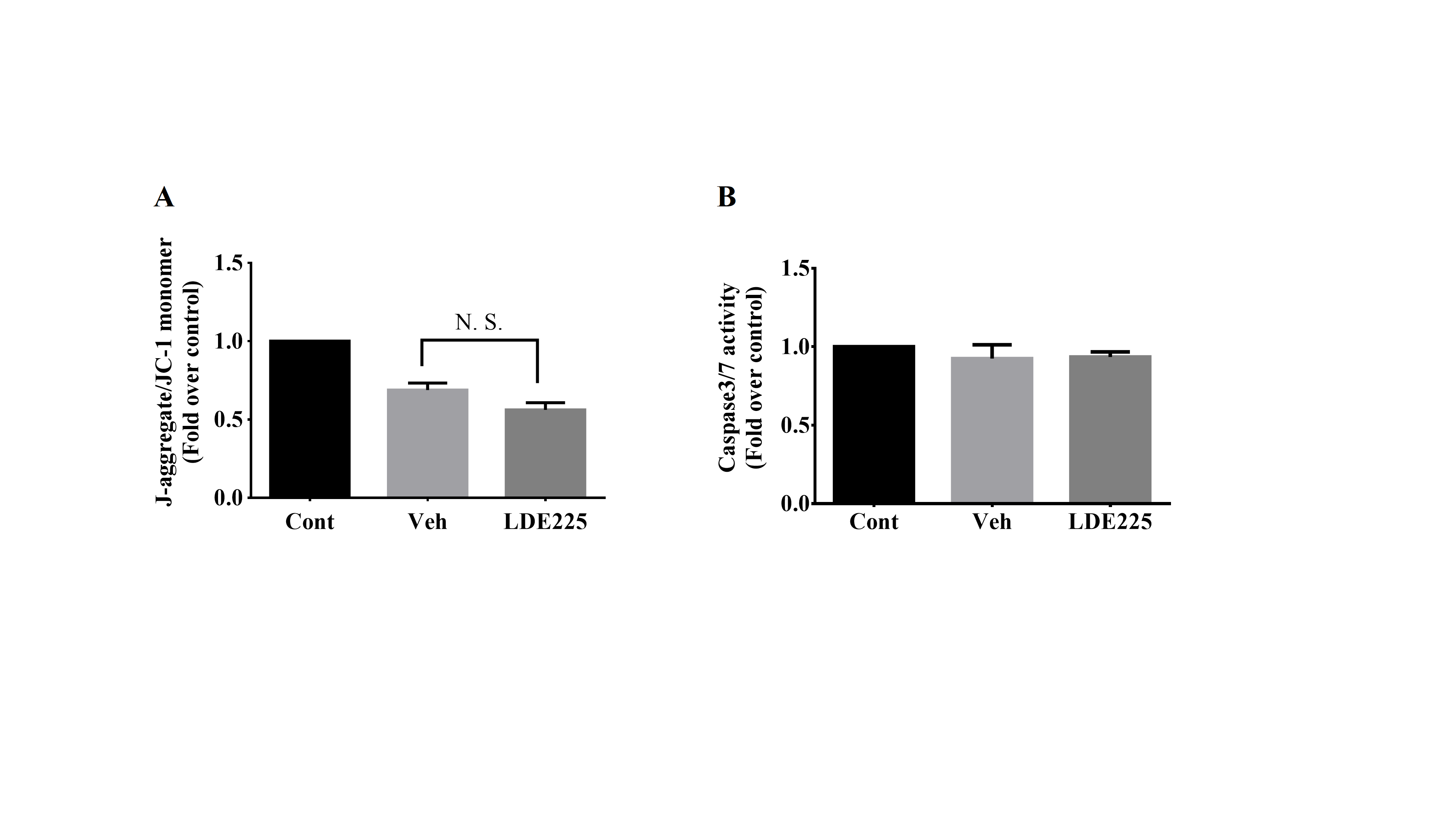

Supplement: Supplemental Figure 3 — LDE225-induced cell death is not mediated primarily through apoptosis. CD133+ cells were treated with LDE225 (25 μM) or vehicle for 48 h. (A) JC-1 assay detect the changes of mitochondria potential which is able to measure early apoptosis. Vehicle (0.1% DMSO) led to mitochondrial depolarization with the decreased ratio of J-aggregate/JC-1 monomer. There was not different from LDE225 (25 μM) treatment. (B) Caspase/Glo assay revealed the activity of Caspase 3/7. There was not different between LDE225 and vehicle treatment. [file Image_3.TIF]

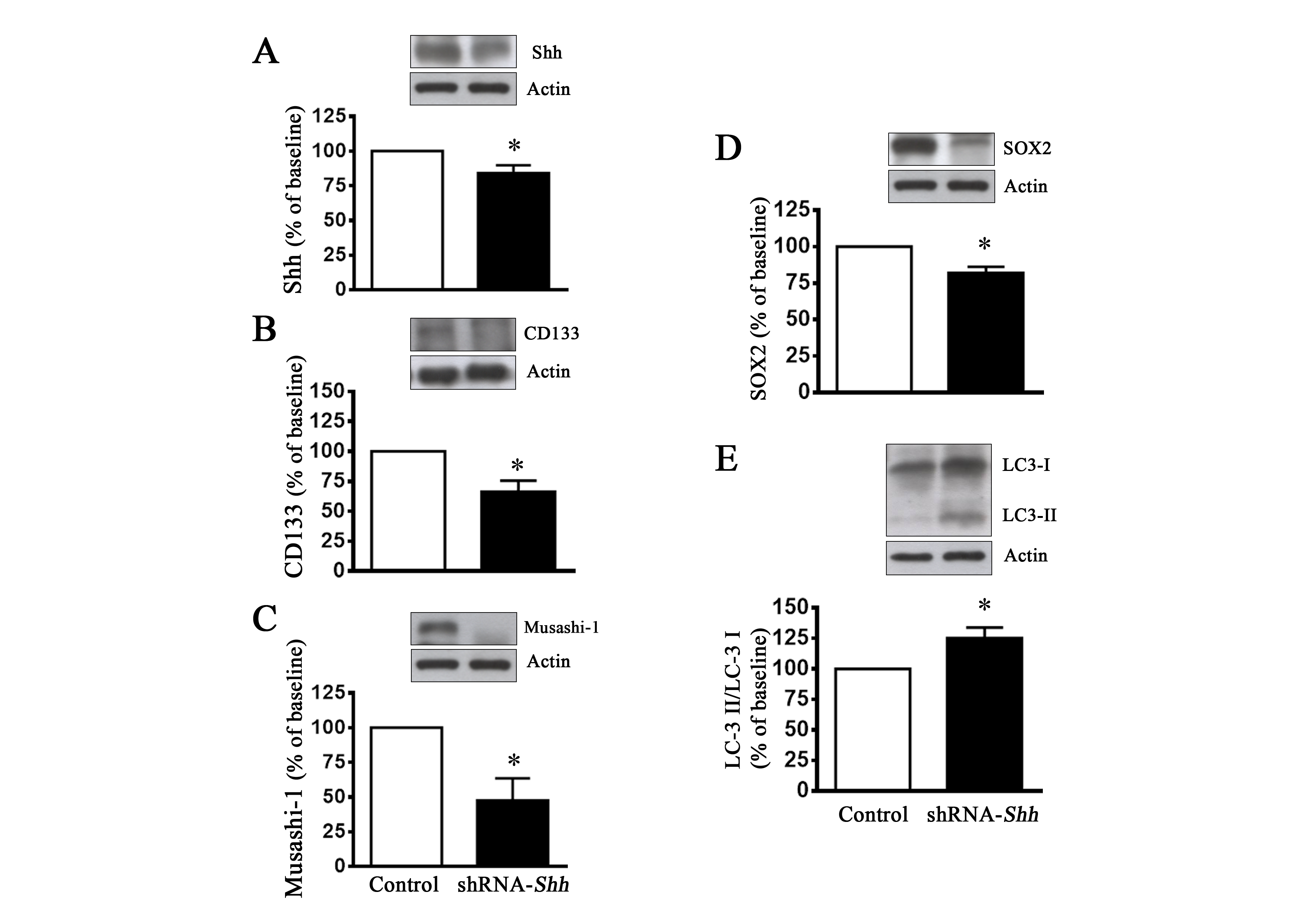

Supplement: Supplemental Figure 4 — The conversion of LC3-I to LC3-II was enhanced in Shh shRNA transfection CD133+-bearing mice. Tumor tissues were collected from Shh shRNA or vector-control transfection CD133+-bearing mice. (A) The efficiency of Shh shRNA-mediated knockdown of Shh was confirmed by western blot analysis. (B–D) The levels of CD133 (B), mushashi-1 (C), and SOX2 (D) were lower in Shh shRNA transfection CD133+-bearing mice. (E) The conversion of LC3-I to LC3-II was enhanced by Shh shRNA transfection. *p < 0.05 vs. control. [file Image_4.TIF]

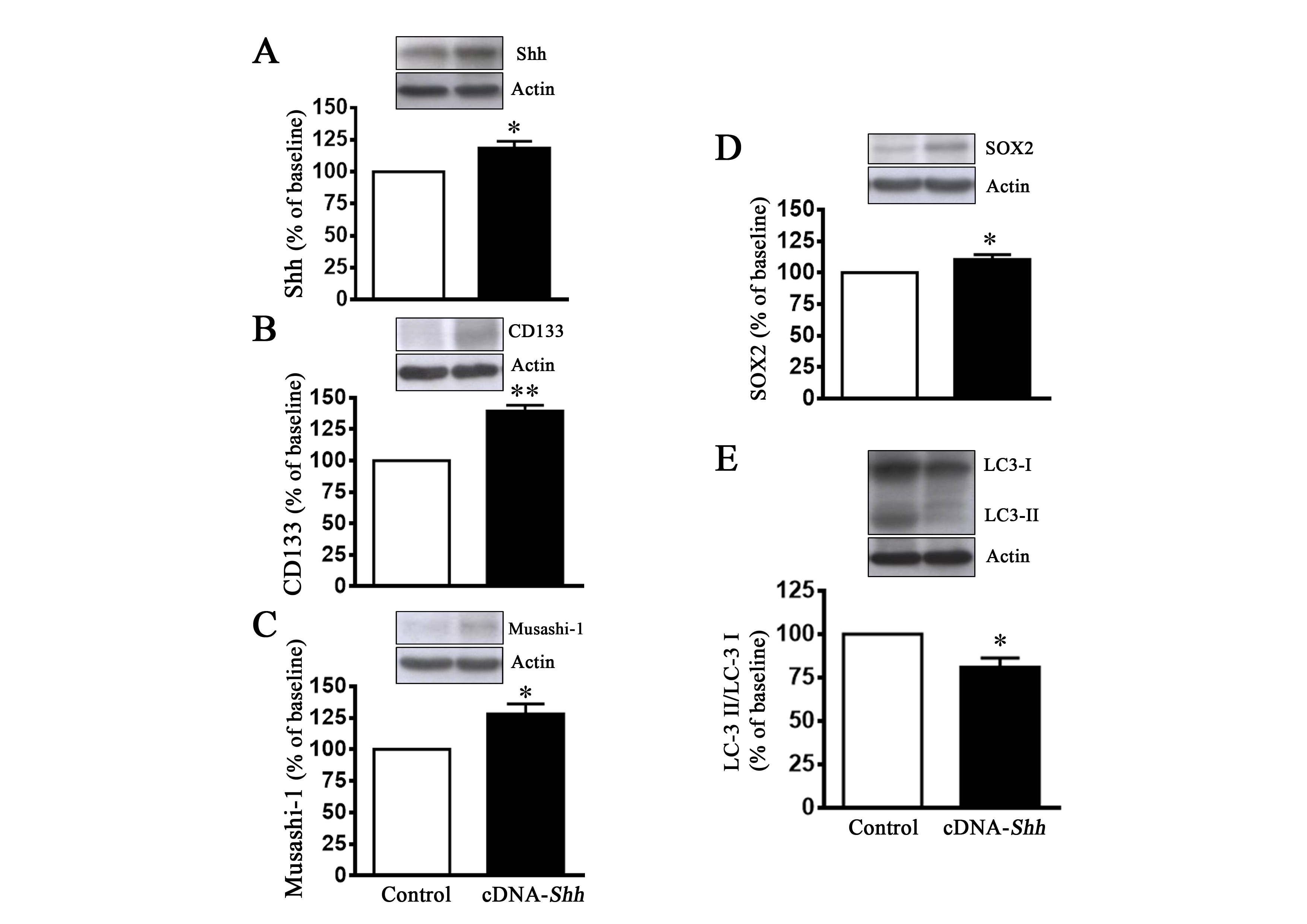

Supplement: Supplemental Figure 5 — The conversion of LC3-I to LC3-II was lower in Shh over-expression GBM-bearing mice. Tumor tissues were collected from LV-Shh or vector-control transfection GBM -bearing mice. (A) The efficiency of LV-Shh-mediated over-expression of Shh was confirmed by western blot analysis. (B–D) The levels of CD133 (B), mushashi-1 (C), and SOX2 (D) were higher in LV-Shh transfection GBM-bearing mice. (E) The conversion of LC3-I to LC3-II was reduced by LV-Shh transfection. *p < 0.05, **p < 0.01 vs. control. [file Image_5.TIF]
